# Latent profiles of work readiness and their associated factors among nursing interns: a cross-sectional study

**DOI:** 10.3389/fmed.2026.1937251

**Published:** 2026-10-16

**Authors:** Yanfang Zhang, Xinxin Xie, Man Wu, Qingqing Lv, Long Zhao, Xiumu Yang

**Affiliations:** 1 School of Nursing, Bengbu Medical University, Bengbu, China; 2 Nursing Department, The First Affiliated Hospital of Anhui Medical University, Hefei, China; 3 Department of Nursing, Tongling Vocational and Technical College, Tongling, China; 4 Department of Nursing, Bozhou Vocational and Technical College, Bozhou, China

**Keywords:** clinical learning environment, generative artificial intelligence literacy, latent profile analysis, nursing interns, Social Cognitive Career Theory, work readiness

## Abstract

**Background:**

Work readiness refers to the comprehensive competencies required for individuals to enter and adapt to the workforce. It is relevant to the transition from nursing student to clinical nurse. However, existing research has paid limited attention to heterogeneity in nursing interns’ work readiness, and few studies have examined associated factors from the perspective of Social Cognitive Career Theory. This study aimed to identify latent profiles of work readiness among nursing interns and examine the associations of generative artificial intelligence (GenAI) literacy and the clinical learning environment with profile membership.

**Methods:**

A cross-sectional study was conducted among 712 final-year nursing interns from three nursing schools in Anhui Province, China, between November 2025 and June 2026. Participants completed the Nursing Practice Readiness Scale, Clinical Learning Environment Questionnaire, and Generative Artificial Intelligence Literacy Scale. Latent Profile Analysis was used to identify distinct work readiness profiles, and multinomial logistic regression was performed to examine factors associated with profile membership.

**Results:**

Three latent profiles of work readiness were identified: low work readiness (*n* = 415, 58.3%), moderate work readiness (*n* = 227, 31.9%), and high work readiness (*n* = 70, 9.8%). The overall multinomial logistic regression model(*χ*^2^ = 467.253, *P* < 0.001) showed that the clinical learning environment, GenAI literacy, academic performance, clinical learning outcomes, and sense of professional belonging were significantly associated with work readiness profile membership (all *P* < 0.05).

**Conclusions:**

Three distinct work readiness profiles were identified among nursing interns. Clinical learning environment, GenAI literacy, academic performance, clinical learning outcomes, and sense of professional belonging were associated with profile membership. These findings highlight the heterogeneity of nursing interns’ work readiness and may inform the development of individualized educational support strategies.

## Introduction

The nursing employment landscape has gradually shifted from a general demand for workforce expansion toward greater emphasis on competency-based recruitment and high-quality job matching (1). Consequently, healthcare institutions have increasingly highlighted the importance of comprehensive competence, clinical adaptability, and workplace preparedness among nursing graduates (2). For nursing interns approaching graduation, the transition from academic learning to professional practice represents a critical developmental stage characterized by both role transformation and employment challenges (3). Traditional academic achievement alone may not fully reflect their ability to adapt to complex clinical environments.

Work readiness is a multidimensional construct reflecting an individual's preparedness in professional knowledge, clinical skills, attitudes, psychosocial characteristics, and workplace adaptability before entering professional practice (4, 5). It is closely related to nursing graduates’ transition to clinical roles and their ability to integrate academic learning with practical competence (6). Previous studies have shown that nursing students may demonstrate different patterns of strengths and weaknesses across clinical skills, professional identity, psychological readiness, and interpersonal competence, suggesting potential heterogeneity in work readiness development (7). Existing research on nursing work readiness has primarily focused on newly graduated or practicing nurses (8, 9), while limited attention has been paid to the heterogeneity and underlying profiles of work readiness among nursing interns during the school-to-work transition period. Latent Profile Analysis (LPA) is an individual-centered statistical approach that can identify unobserved subgroups based on patterns across multiple indicators, providing a useful framework for exploring heterogeneity within complex psychological and behavioral constructs (10). Therefore, identifying distinct work readiness profiles among nursing interns may contribute to a more precise understanding of their developmental characteristics and inform tailored educational support.

The clinical learning environment (CLE) is an important contextual factor connecting nursing education and clinical practice. A supportive CLE provides opportunities for clinical experience, professional feedback, and social support, which have been associated with nursing students’ competency development and work readiness (11–13). However, work readiness is also influenced by individual characteristics. With the rapid development of intelligent healthcare, generative artificial intelligence (GenAI) literacy has emerged as an important competency for future nursing professionals. GenAI literacy extends beyond technical operation and includes understanding intelligent technologies, applying digital tools appropriately, and evaluating ethical issues related to their use (14, 15). Previous studies have suggested that higher AI literacy is associated with improved digital competence, learning capability, and workplace adaptability among nursing students (16, 17).

Social Cognitive Career Theory (SCCT) provides a useful framework for understanding how personal factors and contextual factors interact in career development (18). Within this framework, CLE can be considered a contextual resource that provides learning opportunities and social support, whereas GenAI literacy represents a personal competency that may influence students’ engagement with digital learning resources and clinical environments (19). Although previous studies have examined the relationships between clinical learning environments, digital literacy, and nursing students’ professional development separately (20–22), limited research has investigated how personal and contextual factors jointly relate to different patterns of work readiness. This gap is particularly relevant in the era of intelligent healthcare, where digital competence and clinical learning experiences increasingly intersect (23, 24).

Therefore, guided by SCCT, this study applied Latent Profile Analysis to identify distinct work readiness profiles among nursing interns and examined the associations of clinical learning environment and GenAI literacy, together with other relevant factors, with profile membership. The findings may provide insights into the heterogeneity of nursing interns’ work readiness and inform future research and educational strategies aimed at supporting the transition from nursing education to clinical practice.

## Materials and methods

### Study participants and procedures

A convenience sampling method was used. From November 2025 to June 2026, final-year nursing students from three nursing schools in Anhui Province, China, were recruited. The inclusion criteria were: (1) full-time nursing students scheduled to graduate in 2026; (2) completion of at least 8 months of clinical practicum; (3) being unemployed or actively seeking employment at the time of the survey; and (4) providing written informed consent and voluntarily participating in the study. The exclusion criterion was interruption of clinical practice for 1 month or longer due to illness, personal leave, or other reasons. This criterion was applied because prolonged interruptions may result in differences in clinical exposure and practicum experiences, which could be related to employment-related perceptions and other study variables.

For descriptive cross-sectional research involving quantitative variables, the sample size was calculated using the following formula: *n* = u^2^*_α_*_/2_*σ*^2^/*δ*^2^ (25). In the present study, u*_α_*_/2_ was set to 1.96; referring to previous relevant research (26), the standard deviation *σ* was 49.7, and the allowable error *δ* was 4. The calculated theoretical sample size was 600. Taking a 20% invalid questionnaire response rate into consideration, a total of 750 participants were required for this investigation.

### Data collection

Before the survey, all research team members received standardized training to ensure consistency in participant recruitment, questionnaire administration, and data management. Participants were recruited using convenience sampling from three nursing schools in Anhui Province, China. With assistance from college counselors, eligible final-year undergraduate nursing students were informed of the study purpose, procedures, and survey schedule. Participation was voluntary, and written informed consent was obtained from all participants. Online questionnaires were used as the primary data collection method, supplemented by paper-based questionnaires. Online questionnaires were distributed through WeChat or QQ groups, while paper questionnaires were administered and collected during grade-level class meetings. The first page of each questionnaire explained the study purpose, survey content, data use, and confidentiality measures. Participants provided informed consent before completing the questionnaire. The online platform limited each IP address to one submission to reduce duplicate responses. To ensure data quality, predefined criteria for invalid responses were established before data analysis. Questionnaires were excluded if they had substantial missing responses, showed little response variability (e.g., selecting the same option for most items within a scale), or contained logical inconsistencies among related items. These procedures were used to reduce potentially inattentive or unreliable responses.

A total of 750 questionnaires were collected. After data screening, 38 were excluded according to the predefined criteria, leaving 712 valid questionnaires, with an effective response rate of 94.93%.

## Measurements

### General information questionnaire

Participants’ demographic and individual background characteristics were collected using a self-designed General Information Questionnaire. The variables included gender, residential location, parental career expectations, perceived support from clinical preceptors, student leadership experience (e.g., class or student organization positions), academic performance, motivation for choosing nursing as a major, clinical learning outcomes, and sense of professional belonging. These variables were treated as background characteristics and potential influencing factors in subsequent analyses.

### Nursing practice readiness scale (NPRS)

This scale was originally developed by Kim et al. (26) to assess workforce readiness among nursing interns in terms of knowledge, skills, and attitudes. Zhang et al. (27) adapted and validated the Chinese version, which consists of 35 items across five dimensions: Clinical Judgment and Nursing Performance, Professional Attitude, Patient-Centeredness, Self-Regulation, and Collaborative Interpersonal Relationships. Each item is rated on a 4-point Likert scale ranging from 1 (“strongly disagree”) to 4 (“strongly agree”). The total score ranges from 35 to 140, with higher scores indicating a higher level of nursing practice readiness. In the present study, the NPRS was applied to final-year nursing students before graduation. The overall Cronbach's *α* coefficient was 0.91, with dimension-specific coefficients ranging from 0.83 to 0.92. Confirmatory factor analysis (CFA) showed acceptable model fit (*χ^2^*/df = 1.975, RMSEA = 0.037, CFI = 0.954, TLI = 0.949, NFI = 0.912), supporting the structural validity of the Chinese version of the NPRS in this population.

### Clinical learning environment questionnaire (CLEQ)

The original English version of the CLEQ was developed by AlHaqwi et al. (28) in 2014, consisting of 40 items distributed across five dimensions: clinical case exposure, authenticity of clinical experience, preceptor supervision, patient care organization, and learning capacity. Alnaami et al. (29) later revised the scale into a shortened form. In 2025, Yang et al. (30) completed the Chinese cultural adaptation of the CLEQ. The Chinese version contains 18 items grouped into four dimensions: clinical case exposure, patient care organization, preceptor supervision, and learning motivation. All items are rated on a 5-point Likert scale ranging from 0 (strongly disagree) to 4 (strongly agree). The total possible score ranges from 0 to 72, with higher total scores indicating more positive perceptions of the clinical learning environment. In the present study, the overall Cronbach's *α* coefficient of the scale was 0.876, and the Cronbach's *α* values for each dimension fell between 0.722 and 0.816.

### Generative artificial intelligence literacy scale

This scale was developed by Liu et al. (31), consisting of 38 items, including 5 dimensions: Awakening of GenAI Awareness, Knowledge Construction of GenAI, Development of GenAI competencies, Shaping of GenAI mindset, and Ethical Cognition of GenAI. All items were scored on a 5-point Likert scale (1 = strongly disagree, 5 = strongly agree). The total score ranged from 38 to 190, with higher scores indicating higher levels of individual GenAI literacy. The internal consistency coefficients of each dimension ranged from 0.70 to 0.83. In this study, the overall Cronbach's *α* coefficient was 0.90, and the coefficients of each dimension ranged from 0.68 to 0.75.

### Data analysis

LPA was conducted using Mplus version 8.3 to identify distinct subgroups of participants based on nursing practice readiness. The five dimension scores of the NPRS were entered as continuous indicators, allowing each latent profile to be characterized by a distinct pattern across the five dimensions. Models with one to five profiles were estimated sequentially. The optimal profile solution was determined based on multiple statistical and substantive criteria rather than a single fit index. Model fit was evaluated using the Akaike Information Criterion (AIC), Bayesian Information Criterion (BIC), and sample-size-adjusted Bayesian Information Criterion (aBIC), with lower values indicating better relative fit. Entropy was examined as an indicator of classification quality, with higher values indicating clearer separation among profiles. The Lo–Mendell–Rubin adjusted likelihood ratio test (LMRT) and bootstrap likelihood ratio test (BLRT) were used to compare the k-profile model with the k−1-profile model; a significant result (*P* < 0.05) indicated improved model fit for the k-profile solution (32).

Profile selection also considered profile size, interpretability, and model stability. Solutions containing very small profiles were interpreted cautiously because they may be unstable or difficult to interpret substantively. The final solution was selected based on the overall pattern of fit indices, classification quality, likelihood ratio tests, profile size, and the interpretability and theoretical relevance of the profile-specific NPRS patterns. Profile membership was assigned according to the most likely latent profile. Because this approach does not eliminate classification uncertainty, subsequent regression results were interpreted with caution.

Statistical analyses were performed using SPSS version 27.0. Continuous variables were summarized as mean ± standard deviation or median (interquartile range), as appropriate, and categorical variables as frequencies and percentages. The chi-square test was used to compare categorical variables across latent profiles. For continuous or ordinal variables that did not meet the assumptions of parametric tests, the Kruskal–Wallis H test was used. When the Kruskal–Wallis H test was statistically significant, pairwise comparisons were performed using the Mann–Whitney U test with Bonferroni adjustment. Multinomial logistic regression was subsequently conducted to examine factors associated with membership in the identified latent profiles. Statistical significance was set at *P* < 0.05.

### Ethics statement

This study was approved by the Ethics Committee of Bengbu Medical University (Approval No. K2025-222). All participants were fully informed that their participation was voluntary, all survey responses would remain anonymous, and all personal information would be kept strictly confidential. No potential harm was imposed on any participants throughout the investigation, and the research was conducted in full compliance with the principle of non-maleficence.

## Results

### General information

A total of 712 nursing interns were included, with a mean age of 21.50 ± 0.83 years. The demographic characteristics of the participants are presented in Table 1.

**Table 1:** A univariate analysis of potential types of work readiness for nursing interns.

Variables	Total	C1	C2	C3	*χ*^2^/Z/F	*P*
	(*n* = 712)	(*n* = 415)	(*n* = 227)	(*n* = 70)		
Gender					1.411	0.494
Male	93	57	30	6		
Female	619	358	197	64		
Place of residence					2.181	0.336
Urban	108	59	37	12		
Town	172	97	54	21		
Rural	432	259	136	37		
Parental expectation level					0.172	0.918
Low	216	123	70	23		
Uncertain	285	171	87	27		
High	211	121	70	20		
Support from clinical faculty					66.277	<0.001
Poor	11	7	2	2		
Fair	533	353	150	30		
Good	168	55	75	38		
Holding a position in class/club					0.253	0.881
Yes	366	215	117	34		
No	346	200	110	36		
Academic record					75.347	<0.001
Very Poor	17	14	2	1		
Below Average	59	41	15	3		
Average	359	253	93	13		
Outstanding	277	107	117	53		
Reasons for choosing the nursing major					0.549	0.760
Personal interest	101	60	29	12		
Family arrangement	154	87	49	18		
Academic score constraints	307	186	99	22		
Good employment prospects	150	82	50	18		
Clinical learning Outcomes					53.796	<0.001
Poor	68	50	16	2		
Fair	277	198	66	13		
Good	367	167	145	55		
Sense of professional belonging					32.429	<0.001
Poor	42	31	7	4		
Fair	579	353	183	43		
Good	91	31	37	23		
CLE	23.83 ± 9.00	21.23 ± 7.856	26.53 ± 8.284	30.51 ± 11.429	53.66	<0.001
Gen AI Literacy	132.31 ± 14.60	124.88 ± 10.85	139.45 ± 11.18	153.16 ± 11.83	265.479	<0.001

C1 = Low work readiness profile; C2 = Moderate work readiness profile; C3 = High work readiness profile.

### Latent profiles of work readiness among nursing interns

Latent profile models were estimated using the five dimension scores of work readiness as continuous indicators. Models with one to five profiles were estimated sequentially. The fit indices for the candidate models are presented in Table 2. Model selection was based on the overall pattern of AIC, BIC, and aBIC values, entropy, likelihood ratio tests, profile sizes, and substantive interpretability. The 5-profile model had the lowest AIC, BIC, and aBIC values among the candidate models; however, the LMR test was not statistically significant (*P* > 0.05), indicating that the 5-profile model did not provide a statistically significant improvement in fit compared with the 4-profile model. Therefore, the 5-profile solution was not retained. The 4-profile model showed a statistically significant LMR test (*P* < 0.05), indicating improved fit relative to the 3-profile model. However, one profile comprised only 6.5% of the sample, raising concerns about the stability and substantive interpretability of this solution. Thus, despite its favorable statistical fit, the 4-profile model was not retained. The 3-profile model demonstrated relatively favorable entropy and a more balanced distribution of participants across profiles. Considering the information criteria, likelihood ratio tests, classification quality, profile sizes, and substantive interpretability, the 3-profile solution was considered the most appropriate solution for the present data and was retained for subsequent analyses.

**Table 2:** Fit indices of latent profile models for work readiness Among nursing interns (*n* = 712).

Profile	K	AIC	BIC	aBIC	Entropy	LMR	BLRT	Proportion
1	10	19,632.934	19,678.615	19,646.862	-	-	-	-
2	16	19,171.898	19,244.987	19,194.183	0.812	<0.001	<0.001	0.760/0.240
3	22	19,016.919	19,117.416	19,047.561	0.864	<0.001	<0.001	0.583/0.319/0.098
4	28	18,957.440	19,085.346	18,996.439	0.849	0.013	<0.001	0.095/0.065/0.570/0.270
5	34	18,940.862	19,076.177	18,988.218	0.862	0.061	<0.001	0.017/0.250/0.569/0.097/0.067

### Naming of each latent category

Figure 1 illustrates the mean scores across the five dimensions of work readiness across the three latent profiles derived from the optimal 3-profile model. Significant between-profile differences in mean scores were detected for four out of five work readiness dimensions (*P* < 0.05). No statistically significant difference was only observed for the professional attitude dimension between Profile C2 and Profile C3. In addition, the overall score trends across all dimensions were consistent among the three subgroups. The “low”, “moderate”, and “high” labels are purely statistical, referring only to relative score rankings of the latent profiles rather than absolute standards. Profile C1 consisted of 415 nursing interns, accounting for 58.3% of the total sample. Participants in this profile presented lower scores on all work readiness dimensions relative to C2 and C3, which was labelled the low work readiness profile. Profile C2 included 227 participants (31.9% of the sample) and was defined as the moderate work readiness profile. Profile C3 contained 70 nursing interns, representing 9.8% of all respondents. This subgroup exhibited markedly higher scores across all work readiness dimensions compared with C1 and C2, and was categorized as the high work readiness profile(in Figure 1, Table 3).

**Figure 1:**
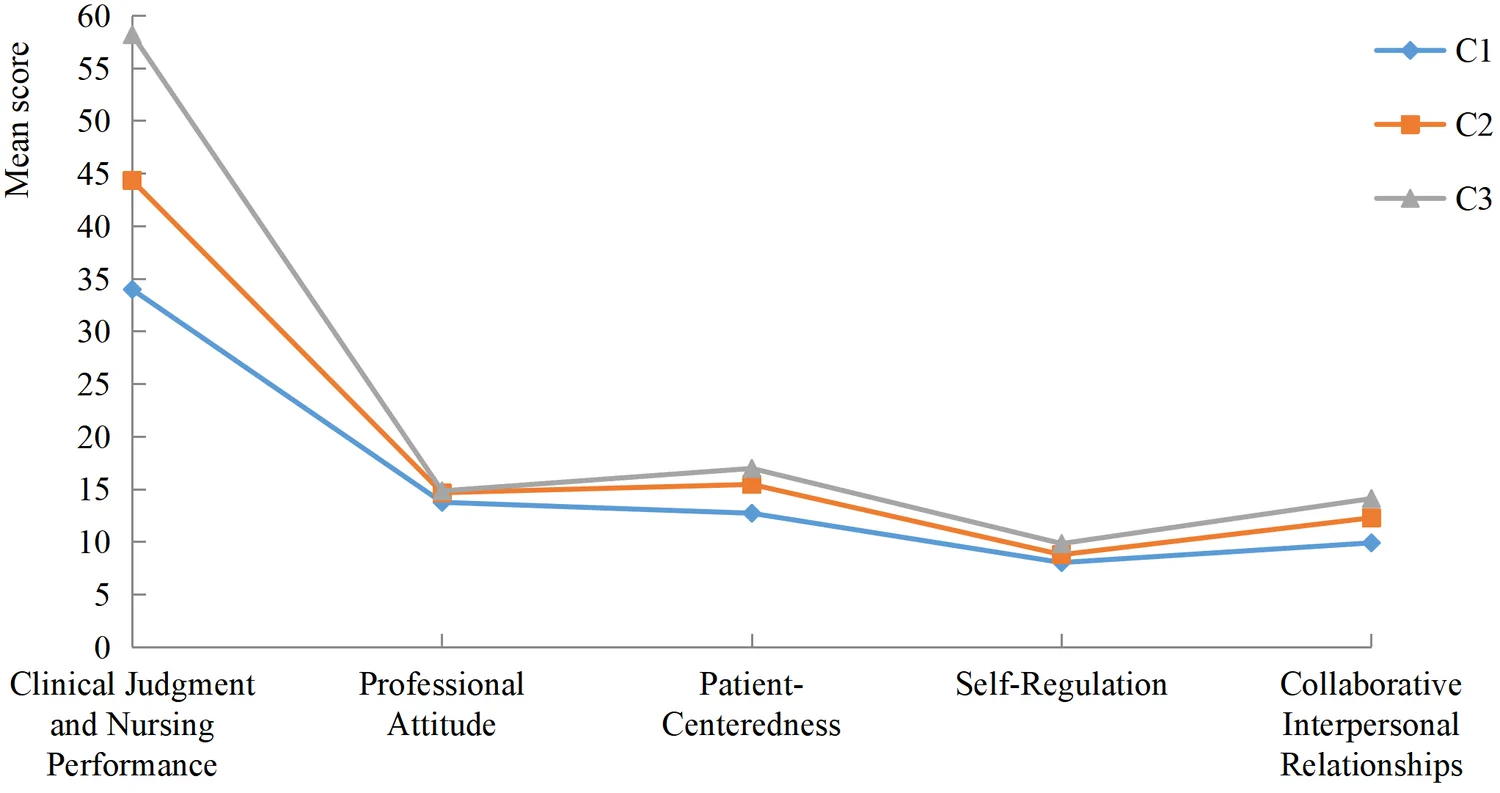
Mean distribution of work readiness across three latent profiles among nursing interns.

**Table 3:** Numerical means and uncertainty estimates for each profile.

variables	C1			C2			C3		
	Means	SE	95% CI	Means	SE	95% CI	Means	SE	95% CI
Clinical judgment and nursing performance	33.992	0.214	33.707，34.547	44.346	0.414	43.376，44.999	58.211	0.665	56.697，59.303
Professional attitude	13.764	0.195	13.391, 14.156	14.682	0.318	14.175, 15.320	14.848	0.509	13.767, 15.764
Patient-centeredness	12.725	0.148	12.456, 13.037	15.467	0.233	15.052, 15.891	16.986	0.420	16.320, 17.835
Self-regulation	8.039	0.144	7.774, 8.338	8.789	0.197	8.425, 9.137	9.843	0.316	9.345, 10.485
Collaborative interpersonal relationships	9.912	0.116	9.725, 10.180	12.296	0.194	11.920, 12.619	14.122	0.279	13.641, 14.647

### Univariate analysis of latent work readiness profiles

Chi-square tests and rank-sum tests were performed to compare the distribution of individual background factors across distinct latent work readiness profiles of nursing interns. The corresponding results are displayed in Table 1.

### Multinomial logistic regression of latent work readiness profiles

A multinomial logistic regression model was constructed, with latent work readiness profiles defined as the dependent variable and Profile C1 serving as the reference group. Covariates included in the final regression model were CLE, GenAI literacy, preceptor support, academic record, sense of professional belonging, and clinical learning outcomes. The overall multinomial logistic regression model was statistically significant (*χ*^2^ = 467.253, *P* < 0.05) (in Table 4).

**Table 4:** Multinomial logistic regression analysis of influencing factors of work readiness (*N* = 712).

Variables	*B*	SE	*Wald χ^2^*	*P*	*OR*	95%CI
C2 VS C1						
Intercept	−21.405	2.094	104.527	<0.001		
CLE	0.034	0.014	6.400	0.011	1.035	1.008, 1.063
Gen AI Literacy	0.149	0.015	103.694	<0.001	1.161	1.128, 1.195
Clinical learning Outcomes (ref:Poor)						
Fair	1.435	0.443	10.519	0.001	4.201	1.765, 10.003
Good	0.498	0.244	4.158	0.041	1.645	1.020, 2.654
Sense of professional belonging (ref:Poor)						
Fair	1.028	0.615	2.793	0.095	2.796	0.837, 9.337
Good	0.569	0.350	2.643	0.104	1.766	0.890, 3.506
Academic record (ref:Outstanding)						
Very poor	−2.172	0.884	6.036	0.014	0.114	0.020, 0.644
Below average	−0.904	0.393	5.305	0.021	0.405	0.188, 0.874
Average	−0.920	0.221	17.261	<0.001	0.399	0.258, 0.615
C3 VS C1						
Intercept	−42.293	3.749	127.26	<0.001		
CLE	0.042	0.021	4.060	0.044	1.043	1.001, 1.086
Gen AI Literacy	0.279	0.025	128.717	<0.001	1.321	1.259, 1.387
Clinical learning Outcomes (ref:Poor)						
Fair	2.124	0.940	5.107	0.024	8.365	1.326, 52.781
Good	1.408	0.473	8.868	0.003	4.090	1.618, 10.334
Sense of professional belonging (ref:Poor)						
Fair	4.027	1.003	16.138	<0.001	56.106	7.865, 400.258
Good	1.396	0.553	6.367	0.012	4.039	1.366, 11.946
Academic record (ref:Outstanding)						
Very poor	−1.830	1.283	2.034	0.154	0.160	0.013, 1.984
Below average	−2.042	0.867	5.546	0.019	0.130	0.024, 0.710
Average	−2.034	0.419	23.601	<0.001	0.131	0.058, 0.297

C1 = Low work readiness profile; C2 = Moderate work readiness profile; C3 = High work readiness profile.

A multinomial logistic regression model was constructed with latent work readiness profile membership as the dependent variable, with Profile C1 serving as the reference category. Covariates were selected based on their theoretical relevance to nursing practice readiness and their potential associations identified in the preliminary analyses. The final regression model included CLE, GenAI literacy, clinical preceptor support, academic performance, sense of professional belonging, and clinical learning outcomes.

The results demonstrated that the clinical learning environment was a significant positive predictor of C2 (OR = 1.035, 95% CI: 1.008–1.063), indicating that each one-unit increase in clinical learning environment scores increased the likelihood of being classified into category C2 rather than C1. Generative AI literacy also served as a robust positive influencing factor for C2 classification (OR = 1.161, 95% CI: 1.128–1.195), suggesting that higher generative AI literacy was significantly associated with greater odds of achieving a higher level of work readiness. With poor clinical learning outcomes as the reference, participants with fair clinical learning outcomes showed significantly higher odds of being categorized into C2 (OR = 4.201, 95% CI: 1.765–10.003). Similarly, participants with good clinical learning outcomes were more likely to fall into category C2 (OR = 1.645, 95% CI: 1.020–2.654). Participants with very poor academic records presented significantly lower odds of being classified into C2 (OR = 0.114, 95% CI: 0.020–0.644). Consistently, below-average academic performance (OR = 0.405, 95% CI: 0.188–0.874) and average academic performance (OR = 0.399, 95% CI: 0.258–0.615) were both significant negative predictors of higher-level work readiness classification, indicating that better academic performance corresponded to higher work readiness in the study sample.

Regarding the comparison of C3 versus C1, consistent predictive trends were observed for most variables. The clinical learning environment significantly positively predicted C3 classification (OR = 1.043, 95% CI: 1.001–1.086), Generative AI literacy remained a powerful positive predictor for C3 classification (OR = 1.321, 95% CI: 1.259–1.387). Using poor clinical learning outcomes as the reference, fair (OR = 8.365, 95% CI: 1.326–52.781) and good clinical learning outcomes (OR = 4.090, 95% CI: 1.618–10.334) were significantly associated with increased odds of C3. sense of professional belonging showed significant predictive effect on C3 classification, with fair (OR = 56.106, 95% CI: 7.865–400.258) and good professional belonging (OR = 4.039, 95% CI: 1.366–11.946). With outstanding academic performance as the reference, poorer academic performance was significantly linked to lower odds of C3 classification. Very poor (OR = 0.160, 95% CI: 0.013–1.984), below-average (OR = 0.130, 95% CI: 0.024–0.710), and average academic performance (OR = 0.131, 95% CI: 0.058–0.297) all acted as significant negative predictors for C3 grouping.

## Discussion

The latent profile analysis identified three distinct work readiness profiles among nursing interns: low, moderate, and high work readiness profiles. These profiles demonstrated different patterns across psychological cognition, clinical competence, and sense of professional belonging. The low work readiness profile (C1) represented the largest subgroup, accounting for 58.3% of the sample. Previous studies have indicated that nursing interns commonly experience transition difficulties during the transition from school to clinical practice, including role uncertainty, challenges in knowledge application, and reduced confidence (33). The consistently low scores across dimensions in C1 suggest that this subgroup may face greater challenges in adapting to clinical practice and developing work readiness. This finding highlights the potential value of strengthening foundational clinical training, simulation opportunities, and individualized guidance before graduation (34, 35).

The moderate work readiness profile (C2) accounted for 31.9% of participants, consistent with previous findings reported by Liu et al. (36). This subgroup showed intermediate levels across dimensions but demonstrated relatively uneven development across competencies. Compared with the low-readiness group, these students may have acquired basic clinical abilities but may require further support in advanced competencies, such as clinical reasoning, emergency response, and interdisciplinary collaboration. Previous research has suggested that supportive clinical learning environments and effective preceptor guidance are important contextual resources during competency development (35). Therefore, educational approaches such as case-based learning, simulation training, and increased opportunities for supervised clinical decision-making may be considered to further support students in this profile (37, 38).

The high work readiness profile (C3) represented 9.8% of participants, which was comparable with previous findings by Qin et al. (7). Students in this profile demonstrated relatively higher levels of work readiness, which may be associated with stronger professional identification, autonomous learning behaviors, and richer clinical experiences (39). Previous studies have also reported associations between work readiness, professional commitment, and critical thinking ability (40). However, standardized training approaches may not fully address the diverse developmental needs of high-readiness students. Providing opportunities such as peer mentoring or clinical teaching assistance may help these students further develop their competencies while contributing positively to the learning environment of other nursing interns (41).

The present study found that a more supportive clinical learning environment was associated with higher work readiness profiles among nursing interns. This finding is consistent with previous evidence that clinical learning environments, together with opportunities for skill development, clinical experience, and clinical reasoning, are associated with nursing students’ occupational readiness and perceived competence (42). From the perspective of SCCT, contextual affordances can interact with personal and social-cognitive factors to shape learning and career-related behaviors (18, 19). A supportive clinical environment, characterized by structured preceptorship, adequate learning resources, and an inclusive atmosphere, may provide opportunities for successful learning experiences and social support, which may be associated with stronger self-efficacy and more positive outcome expectations (43). Conversely, unsupportive environments may function as contextual barriers and be associated with greater occupational stress and less favorable learning experiences (44). Although these mechanisms were not directly examined in this study, clinical training institutions may consider strengthening preceptorship systems, standardized clinical guidance, and supportive learning climates to facilitate work readiness.

Higher GenAI literacy was also associated with membership in the moderate- and high-work-readiness profiles. Within the SCCT framework, GenAI literacy may represent a personal resource that interacts with contextual learning opportunities and social-cognitive factors. Nursing interns with greater GenAI literacy may be better able to access clinical information, organize learning resources, and use digital tools for clinical learning, which may be associated with stronger digital self-efficacy and more positive outcome expectations (19, 43). However, the present cross-sectional findings do not establish a causal relationship or directly test these mechanisms. Nursing educators may therefore consider incorporating appropriate GenAI-related activities into curricula and clinical training to support students’ digital learning competencies.

Better clinical learning outcomes were positively associated with higher work readiness profiles. Students reporting more favorable learning outcomes may also have greater confidence in applying knowledge and skills in clinical situations, which may be related to higher work readiness. Nevertheless, these mechanisms were not directly assessed in the present study (7, 42). No statistically significant difference was observed between participants with poor and good clinical learning outcomes. This finding should not be interpreted as evidence of equivalence, as the relatively small and unevenly distributed poor-outcome group may have reduced statistical power. Clinically, regular formative assessment, structured feedback, case-based review, and targeted practice may be considered to address competency gaps (45).

A higher sense of professional belonging was associated with membership in C3 rather than C1, whereas no statistically significant association was observed in the C2 versus C1 comparison. From an SCCT perspective, professional belonging may represent a personal and social-contextual resource related to students’ perceptions of their professional identity, self-efficacy, and career expectations (46, 47). However, the different findings across profile comparisons do not provide sufficient evidence for a linear, threshold, or non-linear effect of professional belonging on work readiness. The nonsignificant C2 versus C1 comparison should likewise be interpreted as an absence of a detected association rather than evidence that professional belonging is unrelated to work readiness. Further longitudinal research is needed to clarify how professional belonging interacts with personal and contextual factors. Nursing schools and clinical training institutions may consider professional identity education, mentorship, and humanistic care as potential approaches to supporting students’ professional belonging and work readiness.

Academic performance showed a differentiated pattern across work readiness profiles. Because work readiness encompasses theoretical knowledge, clinical reasoning, self-efficacy, communication, adaptability, and other non-cognitive competencies (48), academic performance may capture only one aspect of this broader construct. Thus, strong academic performance may not necessarily correspond to higher overall work readiness when students differ in clinical adaptation, communication, teamwork, or situational decision-making (49). Conversely, students with moderate or lower academic performance may still demonstrate relatively favorable work readiness in the presence of supportive clinical experiences or other contextual resources (50). For those with lower academic performance but relatively higher work readiness, opportunities to strengthen theoretical knowledge while maintaining clinical engagement may be useful. Flipped classrooms, simulation-based learning, and integrated theory–practice activities may be considered as potential approaches (51), although their effects on work readiness require further empirical evaluation.

### Limitations

This study has several limitations. First, participants were recruited from only three nursing institutions in Anhui Province, China, using convenience sampling, which may limit sample representativeness and the generalizability of the findings. Future multicenter studies with more diverse samples are needed to validate these results. In addition, the exclusion of students who interrupted clinical practice for one month or longer may have introduced selection bias, as these students may have different clinical learning experiences. Second, the study relied on self-reported questionnaires, which may be affected by recall bias, social desirability bias, and common-method bias. Although standardized procedures were implemented, future studies should consider incorporating objective indicators or multiple data sources. Moreover, the NPRS was originally developed for newly employed nurses, and further validation among nursing students is warranted. Third, both online and paper-based questionnaires were used, and differences in survey modality may have influenced response patterns. Future studies using consistent data collection methods are recommended. In addition, latent profile classification and subsequent regression analyses may be affected by classification uncertainty and small subgroup sizes, which could result in unstable estimates and wide confidence intervals. Finally, although several relevant factors were included based on the SCCT framework, some potential confounders, such as family background, previous healthcare experience, and characteristics of clinical placements, were not examined. Future longitudinal studies incorporating additional personal, contextual, and social-cognitive factors are needed to further understand nursing students’ work readiness.

## Conclusion

This observational cross-sectional study used latent profile analysis to identify heterogeneous patterns of work readiness among nursing interns and examine factors associated with profile membership. Three distinct latent profiles were identified, including low, moderate, and high work readiness profiles. Clinical learning environment, GenAI literacy, academic performance, clinical learning outcomes, and sense of professional belonging were associated with work readiness profile membership. These findings highlight the heterogeneity of work readiness among nursing interns and suggest that nursing educators and administrators may consider students’ individual characteristics, learning experiences, and contextual factors when developing educational support strategies. Future longitudinal and intervention studies are needed to clarify temporal relationships and evaluate educational strategies for supporting the development of nursing interns’ work readiness.

## Data Availability

The datasets presented in this study can be found in online repositories. The names of the repository/repositories and accession number(s) can be found in the article/Supplementary Material.
